# Point-of-care diagnostic (POCD) method for detecting *Bursaphelenchus xylophilus* in pinewood using recombinase polymerase amplification (RPA) with the portable optical isothermal device (POID)

**DOI:** 10.1371/journal.pone.0227476

**Published:** 2020-01-14

**Authors:** Deokjea Cha, Dongsoo Kim, Wonil Choi, Sungjun Park, Hyerim Han

**Affiliations:** 1 Division of Forest Insect Pests & Diseases, National Institute of Forest Science, Dongdaemun, Seoul, Korea; 2 Division of Forest Ecology & Climate Change, National Institute of Forest Science, Dongdaemun, Seoul, Korea; 3 2NCBio, Seo-gu, Daejeon, Korea; Universidade Nova de Lisboa, PORTUGAL

## Abstract

The pinewood nematode (PWN), *Bursaphelenchus xylophilus*, is a causative agent of pine wilt disease (PWD). To date, although several molecular diagnostic methods have been developed, rapid on-site diagnostic tools for detecting PWN in pinewood are limited. In this study, a point of care diagnostic (POCD) method for detecting PWN in pinewood using recombinase polymerase amplification (RPA) assay was developed. This method comprises quick gDNA extraction buffer (DAP buffer) for the direct extraction of gDNA of PWN from pinewood and a battery-mounted portable optical isothermal device (POID) for the detection of PWD in the field. The RPA assay can distinguish between the PWN and its conspecies which exist in pinewood and can complete diagnostic procedures within 25 min in the field. Moreover, the RPA assay can detect PWN in old wood samples in both natural and stored conditions. The POCD-RPA assay to detect PWN will be useful for epidemiological investigations in the field as well as for quarantine processes in the wood trade.

## Introduction

The rapid and accurate diagnosis of insect pests and pathogens is an essential requirement for agricultural and forest pest management [1]. Early detection and response are the best strategies for managing invasive species [2]. Among the invasive species, sufficient diagnostic methodologies are required to detect tiny or cryptic species. For example, diagnostic methods for the pinewood nematode (PWN), *Bursaphelenchus xylophilus*, which kills pine trees and inhabits the pinewood, are being intensively developed for the early detection of this nematode.

Pine wilt disease (PWD) is caused by the mutual interaction between the PWN and its vector, native *Monochamus* beetles. Because the nematode inhabits the pinewood and tracheal systems of vectors [3], its diagnosis in the field was interactively conducted by observing the symptoms of dead trees [4]. This method is dependent on the experience of the observers, and trees infected with PWN without symptoms are not detected. The importance of the asymptomatic carrier in the spread of PWN has been reported through field observations [5].

The PWN is native to North America and is a major threat to pine trees in Asia. The introduction of the PWN has also been reported in Western European countries, such as Portugal [6] and Spain [7], which are at a considerable distance from Far East Asia. The first study to report PWD in pine forests was conducted in the early 1900s in Japan. Later, similar studies were conducted in such as China [8], Taiwan [9], and South Korea [10], by human-mediated activities [4]. The rising global prevalence of PWD might be due to the increased trade between countries in recent decades [11,12]. Therefore, the rapid quarantine and accurate diagnosis of PWD is a prerequisite to prevent its transmission between countries.

To date, the traditional diagnosis of PWD has mainly been conducted using morphological characteristics to identify the presence or absence of *B*. *xylophilus* from the suspected pine tree through microscopy [13]. However, various nematodes coexist in pine trees, including *Bursaphelenchus* spp., which are non-pathogenic but are similar in morphology [14]. For this reason, skilled nematode expertise is required to diagnose PWD, which can take a long time to implement.

As a result, many previous studies have laid the groundwork for the easy diagnosis of PWN using a variety of molecular diagnostic methods (e.g. PCR, RFLP, Real-time PCR, LAMP; loop-mediated isothermal amplification, RPA; recombinase polymerase amplification) [15–20]. These molecular diagnostic methods for the detection of PWN are relatively easy and accurate for non-experts. Despite the advantages, until now these methods have been difficult to implement in the field because they are time-consuming and require a specialized device as well as a procedure for extracting genomic DNA (gDNA) from *B*. *xylophilus*, which is isolated from pinewood using Baermann funnel methods. To overcome these drawbacks, several studies on molecular detection methods have been conducted to directly extract gDNA from pinewood infected with *B*. *xylophilus* [21,22].

A commercial kit for the detection of PWN using the LAMP method has been developed in Japan [16]. Although this is a simplified method for the detection of PWN in pinewood, it takes at least 1.5 hour to confirm the diagnosis result. Moreover, the amplification process of the target gDNA fragments by isothermal polymerase, as well as the extraction of the gDNA of *B*. *xylophilus* in pinewood by proteinase K containing the buffer, requires a specialized thermal device. Unlike other molecular diagnostic methods (e.g., PCR and Real-time PCR), it is possible to obtain false-positives of samples due to cross-contamination by aerosols of the positive sample during high temperature and long amplification times [23]. For these reasons, a LAMP assay for the detection of PWN was difficult to use for on-site diagnosis.

Recombinase polymerase amplification (RPA) assays for detect several pathogens have been developed in recent years [20]. In a previous study, a PWN detection method was developed using a RPA assay with DAP buffer, which could extract gDNA from *B*. *xylophilus* in pinewood. The total procedure time of this method is 30 min, and the method was able to distinguish between *B*. *xylophilus* and *Bursaphelenchus* spp. [24]. However, this RPA assay has two disadvantages. First, it is difficult for non-experts to distinguish the minute degree of fluorescence for positive or negative reactions. Second, the heavy isothermal devices are not portable in the field, which is necessary to implement the RPA assay. Due to these disadvantages, this method is not applicable to field diagnosis.

Therefore, in this study, the following improvements were applied to the RPA assay to detect PWN in pinewood in the field. First, a new pair of primers was designed for the RPA assay to detect *B*. *xylophilus* even in low populations in pinewood samples. Second, the new RPA assay uses a battery-mounted isothermal device with an optical sensor to obtain the absorbance of the fluorescence from the RPA reaction. Thus, the RPA assay can perform in the field. Finally, the experimental procedure was simplified by three steps of assay procedure (Step 1; gDNA extracted from PWN infected pinewood chips by DAP buffer in 10 min, Step 2; amplify the target sequences from *B*. *xylophilus* by RPA in 15 min, Step 3; verify the positive or negative by POID within seconds) so that the new RPA assay does not require skillful experimental experience.

## Materials and methods

### Nematodes and PWN-infected pinewood

*Bursaphelenchus xylophilus* (*Bx*) and five *Bursaphelenchus* spp.: *B*. *thailandae* (*Bt*), *B*. *doui* (*Bd*), *B*. *hylobianum* (*Bh*), *B*. *mucronatus* Asia type (*BmA*), and *B*. *mucronatus* Europe type (*BmE*), were provided by the laboratory of pine wilt disease from the division of forest insect pests & diseases, the National Institute of Forest Science, South Korea. These nematodes were reared on a fungal mat of *Botrytis cinerea* on potato dextrose agar (PDA) media at 25 ± 1°C and 40% humidity for several generations and were identified by morphological characteristics and genetic differences. Pinewood chips were collected from the PWN‐infected pine trees and healthy pine trees (*Pinus densiflora*, located in Mt. Wora, Jinju‐si, Gyeongsangnam‐do, South Korea). PWN-infected pine trees were sampled based on the evidence of symptoms (complete browning of needles) of PWD throughout the entire crown. Pinewood samples were collected by drilling into the trunk 30 cm below and above breast height with a bit with an 8 mm diameter hole to a depth of 100 mm, gathering 20 g per tree and identifying the presence or absence of *B*. *xylophilus* in the pinewood from each tree using the Baermann funnel method. The PWN-infected pinewood samples used in this study consisted of 308 ± 51 (mean ± SD) individuals of *B*. *xylophilus* per 10 g of pinewood. All sampling was authorized by the National Institute of Forest Science.

### Battery-mounted portable optical isothermal device

The portable optical isothermal device (POID) was provided by 2NCBio (Daejeon, South Korea) and is able to set for the amplification temperature from 35–70°C, and to set for the time from 1 min to 1 hour, and to set for an offset value (the setting for zero points) from 50 to 900. In addition, this device can amplify and detect the level of absorbance of samples up to eight at the same time (S1 Appendix).

The POID has four advantages when used in the field. First, it is smaller (150 x 200 x 35 mm) and lighter (400 g) than conventional devices. This feature might be valuable for users in the field where urgent PWD diagnosis is required. Second, the Lithium-ion battery (4,000 mAh) enables stable power supply, so the POID maintains a constant temperature for isothermal amplification reactions. Third, by using UV-LED, MS-L330UVP-365, (wavelength: min 365—max 375 nm, Moksan Electronics, Seongnam, South Korea) with color light sensor, TSC3772, (TAOS, Unterpremstaetten, Austria), this device can precisely measure the level of absorbance from RPA reactions. Finally, the easy operation of the POID could assist beginners who are not familiar with molecular experiments.

### Genomic DNA extraction for nematodes and pinewood samples

Two types of nematode genomic DNA (gDNA) were used for the experiment. One was extracted using a commercial gDNA kit, all nematodes were extracted using the Baermann funnel method, and the other was directly extracted from pinewood using a DAP buffer (20 mM sodium hydroxide, 5% polyethylene glycol 200, and 5% dimethyl sulfoxide). First, for the specificity and limit of detection (LOD) test, pure gDNA was extracted from about 3,000 individual nematodes for each nematode species (*Bx*, *Bt*, *Bd*, *Bh*, *BmA*, and *BmE*) using the QIAgen DNeasy Blood & Tissue kit (Qiagen, Mississauga, ON, Canada). Second, to extract the gDNA from nematodes in pinewood for use in the RPA assay in the field, approximately 100 mg of pinewood chips from PWN-infected or healthy pine trees were placed in a 2 mL tube filled with 1 mL of DAP buffer. Next, the tubes were vigorously mixed and incubated at room temperature (at 20–25°C) for 10 min. During incubation, the tubes were vigorously tapped three times. After incubation, 3 μL of lysate solution was used as the RPA assay template.

### Composition and procedure of the RPA assay

**Fig 1:**
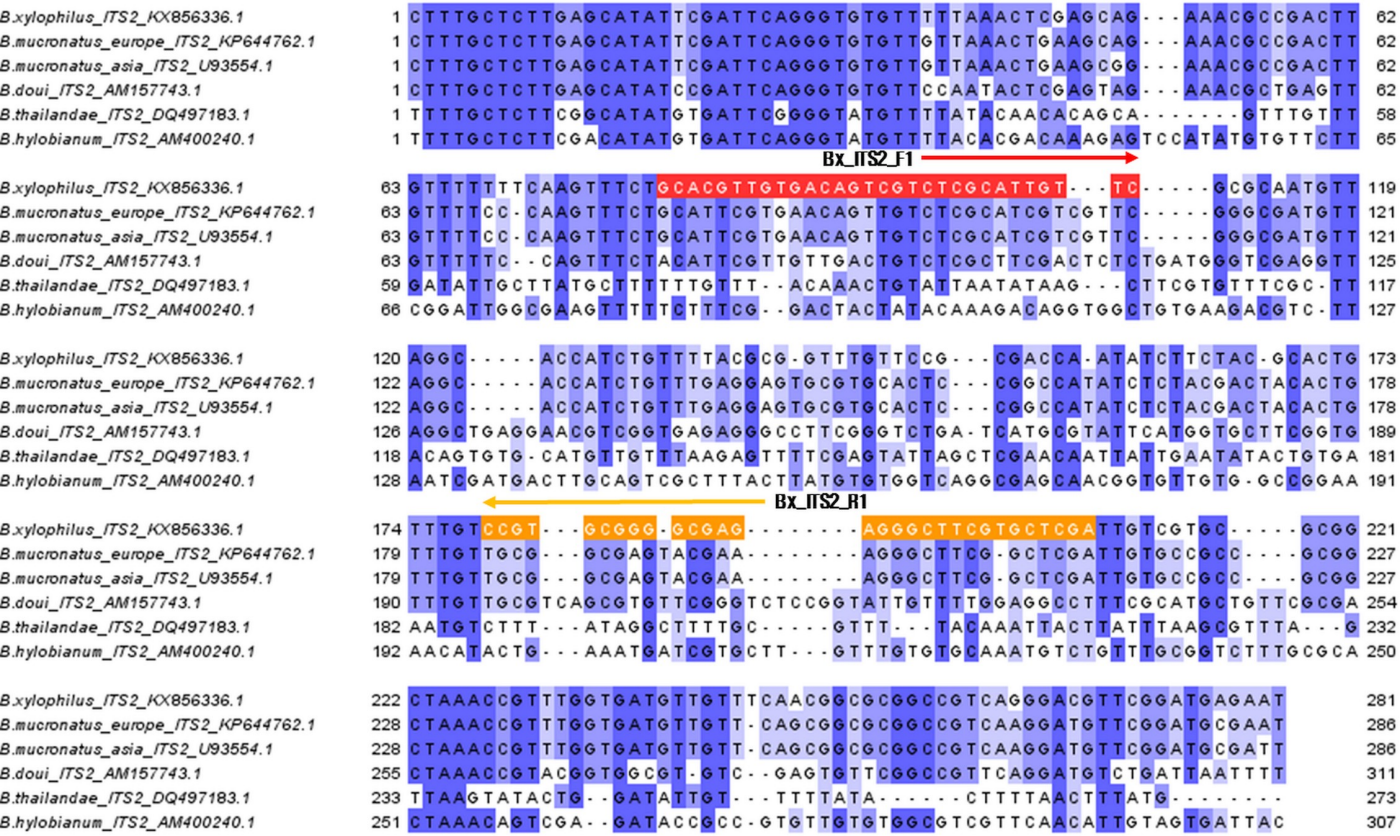
Nucleotide sequence alignment of the internal transcribed spacer 2 (ITS2) from *B*. *xylophilus* (*Bx*), *B*. *mucronatus* Europe type (*BmE*), *B*. *mucronatus* Asia type (*BmA*), *B*. *doui* (*Bd*), *B*. *thailandae* (*Bt*), and *B*. *hylobianum* (*Bh*). Locations of the Bx_ITS2_F1 and Bx_ITS2_R1 primers are marked with a red- and orange-colored box, respectively. Gaps in the sequence alignment are indicated with dots. Sequence identity is indicated with blue-colored gradation.

The primers for the RPA assay were synthesized by Macrogen (Seoul, South Korea) for the amplification of the internal transcribed spacer 2 (ITS2) from *B*. *xylophilus* in the pinewood, and distinguish them from conspecifics. The primers were designed after aligning partial ITS1–5.8S rRNA—ITS2 - 28S rRNA sequences from *B*. *xylophilus* (GenBank accession number: KX856336) and five *Bursaphelenchus* spp.: *B*. *thailandae* (GenBank accession number: DQ497183), *B*. *doui* (GenBank accession number: AM157743), *B*. *hylobianum* (GenBank accession number: AM400240), *B*. *mucronatus* Asia type (GenBank accession number: U93554), and *B*. *mucronatus* Europe type (GenBank accession number: KP644762). The primer sequences Bx_ITS2_F1 5′-Biotin‐GCACGTTGTGACAGTCGTCTCGCATTGTTC‐3′, and Bx_ITS2_R1 5′-FAM-TCGAGCACGAAGCCCTCTCGCCCCGCACGG‐3′ were designed to amplify a 129 base pair fragment exclusively from ITS2 of *B*. *xylophilus* (Fig 1).

**Fig 2:**
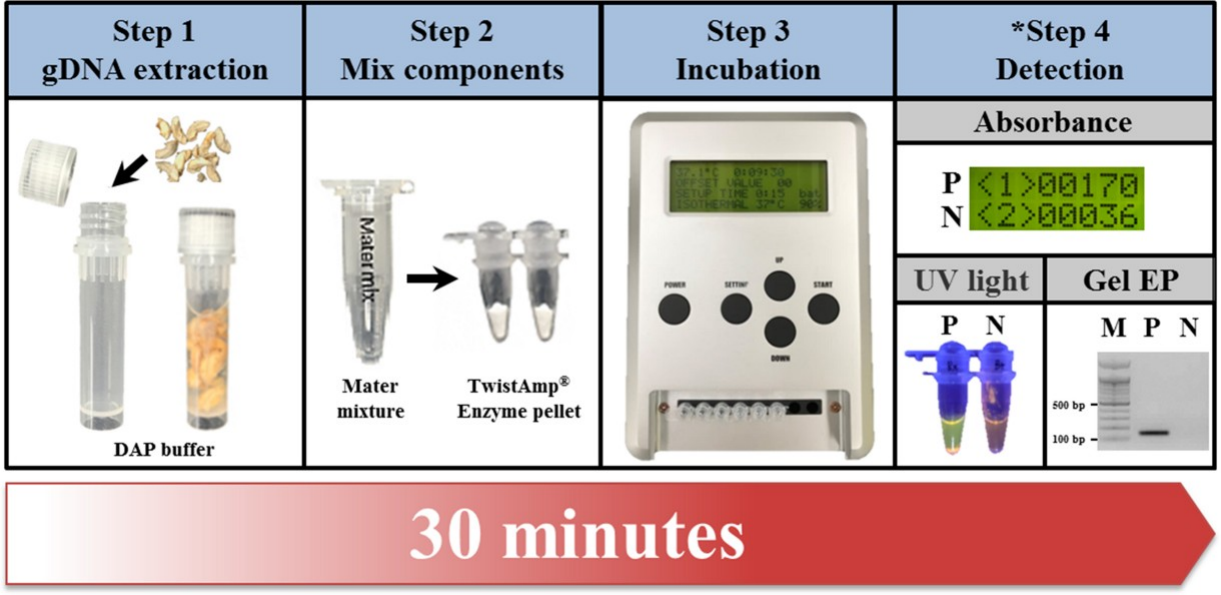
Schematic of the RPA assay for *B*. *xylophilus* in the field with the portable optical isothermal device (POID). P (green-colored) and N (orange-colored) represent the detection results of the absorbance levels (applied non-offset value) and UV light from the positive and negative controls, respectively. *In Step 4, the results of the RPA assay detected by UV light and agarose gel electrophoresis (Gel EP) were presented to help understand the interpretation. M indicates the 100 bp DNA ladder size marker.

The RPA assay was performed using a TwistAmp® basic kit (TwistDX, Cambridge, UK) according to manufacturer's instructions, with some modification. Reagents for the 50 μL of RPA reaction were: 3 μL of lysate solution from the pinewood of PWN-infected or healthy pine trees, or 3 μL of pure gDNA from each nematode as the reaction template, 3.2 μL of the pair of primers (final 0.16 μM each), 11.8 μL of nuclease‐free water untreated with diethyl pyrrolcarbonate (DEPC), 29.5 μL of 1× rehydration buffer, 2.5 μL of magnesium acetate (final 14 mM). In order to be suitable for use in the field, the RPA reaction components, except the TwistAmp® basic enzyme pellet and reaction template (lysate), were composed in the form of mater mixture (total volume of 47 μL) which was durable for use in experiments for up to four months (S2 Appendix). The master mixture with lysate or gDNA was put into the TwistAmp® basic enzyme pellet tube, mixed briefly, and then incubated for 15 min at the optimal temperature (37°C) using the portable optical isothermal device. After incubation, 25 μL of the SYBR green I dye mixture (Lonza, 1:400 nuclease-free water) and 50 μL of the RPA reaction product was mixed, and the absorbance levels were confirmed under ultraviolet (UV) light and by the portable optical isothermal device (POID) (Fig 2).

### The specificity test of the RPA assay

First, the RPA assay was assessed without the gDNA template or RPA primers. In this instance, H_2_O was used as both the gDNA template and primer. In addition, lysate from healthy pine trees was used as the negative control to assess whether the RPA assay alone provokes the amplification of false positives. The zero absorbance point (offset value) of the POID was set according to the results from the negative control test. Experimental repeats were conducted more than 20 times for each negative test.

Next, a specificity test of the RPA assay was conducted to confirm the ITS2 primers that could amplify the specific ITS2 fragment from *B*. *xylophilus* but not from the five *Bursaphelenchus* spp., healthy pine trees, or other insects (*Matsucoccus matsumurae*, *Monochamus saltuarius*, *Monochamus alternatus*, and *Anoplophora chinensis*) using 6 ng of pure gDNA or lysate extracted using DAP buffer from the aforementioned species and pinewood chips. Lastly, the lysates that spiked 60 ng of pure gDNA (the final concentration of gDNA was 6 pg in each RPA reactions) from *B*. *xylophilus* or *Bursaphelenchus* spp. with healthy pinewood from the healthy pine tree that have not *B*. *xylophilus* to verify the amplification results of the pinewood samples that were similar to natural pine trees consisting of various nematodes.

### The limit of detection test of the RPA assay

Similar to the above specificity test of the RPA assay, the limit of detection (LOD) test of the RPA assay was undertaken under optimal condition. First, a ten-fold serial dilution (final concentration from 16 ng to 1.6 fg) of pure gDNA from *B*. *xylophilus* was used as the template to verify the LOD value from the RPA assay in the absence of any interference from the DAP buffer or pinewood which including humic acid, ethanol, polysaccharide, and polyphenol. As above the reason, we designed the pinewood sample LOD test for applied all interferences come from pinewood with DAP buffer using the artificial PWN-infected pinewood samples as RPA assay template. The reason for designed this experiment was that it was difficult to accurately identify the number of *B*. *xylophilus* in the pinewood chips. The artificial PWN-infected pinewood samples were made in the following method. Each six healthy pinewood chip samples were mixed with each pure gDNA of *B*. *xylophilus* before extracted by DAP buffer and then extracted 3 μL of lysates used as RPA assay templates (final concentration from 600 pg to 6 fg).

### Detection of residual environmental DNA from *B*. *xylophilus* in pinewood

In an epidemiological survey conducted in the field, the logs of pine trees in the PWD-infected area and commercial products used in transportation, such as pallets made of pine trees with suspected PWD-infection, might not harbor live *B*. *xylophilus*. However, gDNA from various organisms can remain in the environment for a long time, and such gDNA is called environmental DNA (eDNA). In recent years, several studies regarding the distribution of species by detecting the eDNA present in nature have been conducted. Therefore, two experiments were designed to identify how long eDNA originating from *B*. *xylophilus* remains in the logs of pine trees that were confirmed as PWN-infected. First, three logs of the pine trees with approximately 300 individuals of *B*. *xylophilus* per 10 g of pinewood before the experiment, were incubated at 50°C and 40% humidity until they were not detected by the RPA assay.

In this experiment, the RPA assays were performed 12 times after incubation (at 0, 4, 8, 12, 16, 20, 30, 40, 50, 60, 70, and 80 days). Second, to determine the residual period of eDNA from the logs in nature, four pine tree logs with 50 to 1,500 individuals of *B*. *xylophilus* per 10 g of pinewood were placed in the experimental place that was authorized by the National Institute of Forest Science. In this experiment, the pine tree logs were placed on April 2019, and the RPA assays were conducted from May to August 2019. *B*. *xylophilus* were extracted using the Baermann funnel method and were identified under a microscope for each experiment.

## Results and discussion

### Optimization of the RPA assay for PWN using POID

In a previous study, we confirmed that the RPA assay using primers derived from intergenic spacer sequences (IGS) of *B*. *xylophilus* could detect *B*. *xylophilus* in pinewood in a sufficiently short time [24]. However, this previous method has some disadvantages. First, when the amount of amplification from the RPA reaction was very low, it was not possible to distinguish the negative and positive reactions by visual discrimination under UV light.

Second, although this method was able to confirm results in only 20 min, it was not applicable to the portable isothermal device and subsequent implementation in the field. In contrast, the newly designed RPA assay was optimized to amplify the ITS2 fragment of *B*. *xylophilus* at 37°C for 15 min and can distinguish the negative and positive reactions precisely, even under low concentrations of gDNA, by applying the portable optical isothermal device measuring absorbance values (Fig 2 and S3 Appendix).

### The specificity test of the RPA assay

**Fig 3:**
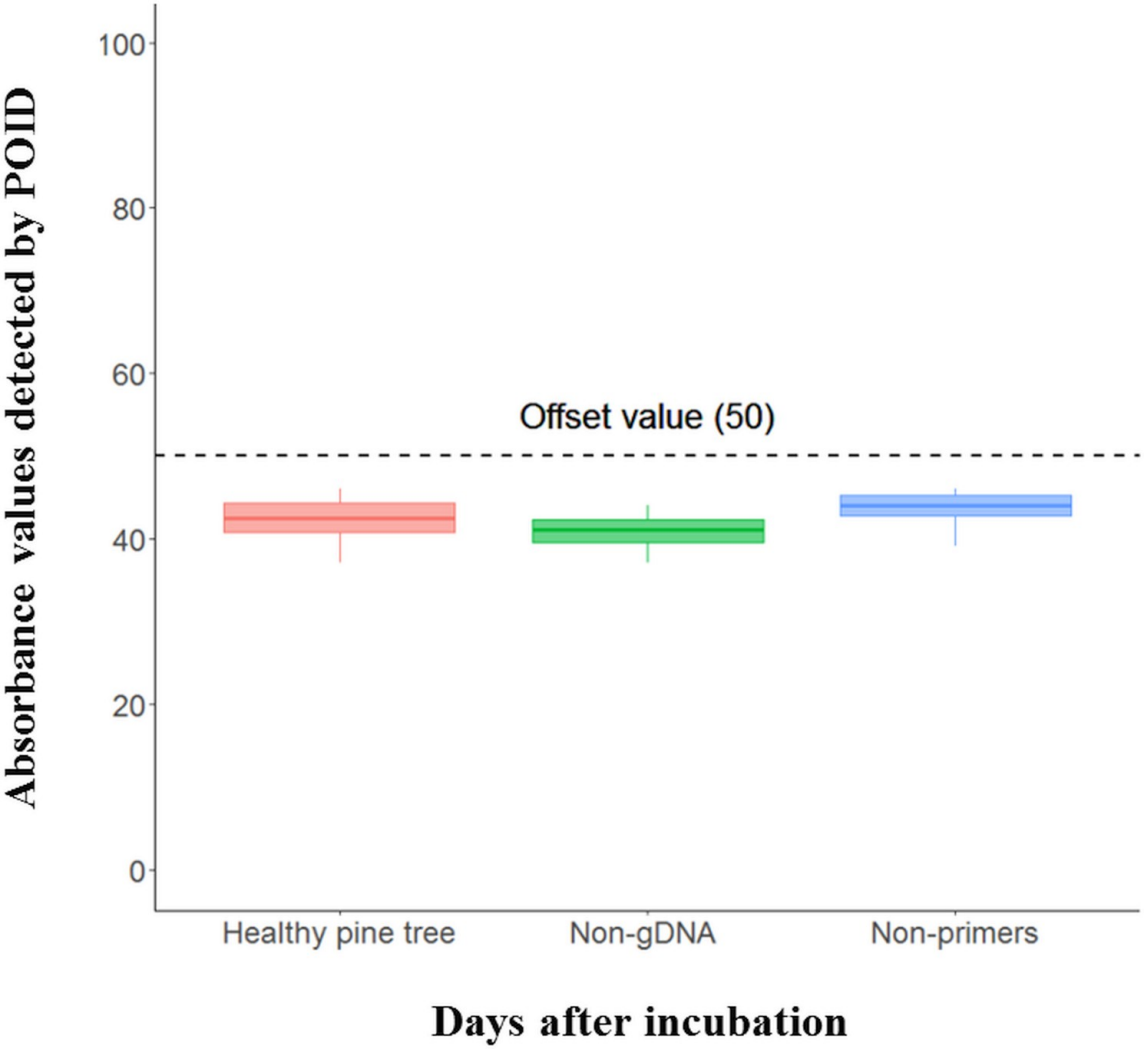
Estimation for the zero absorbance point (offset value) of the portable optical isothermal device (POID) of the RPA assay for the detection of *B*. *xylophilus* in pinewood. Healthy pine tree indicates that the used gDNA template was lysate extracted by the DAP buffer from the healthy pine tree. The non-gDNA and non-primers represent that the RPA assay used H_2_O instead of gDNA or primers. The black dashed line represents the offset value of the RPA assay.

**Fig 4:**
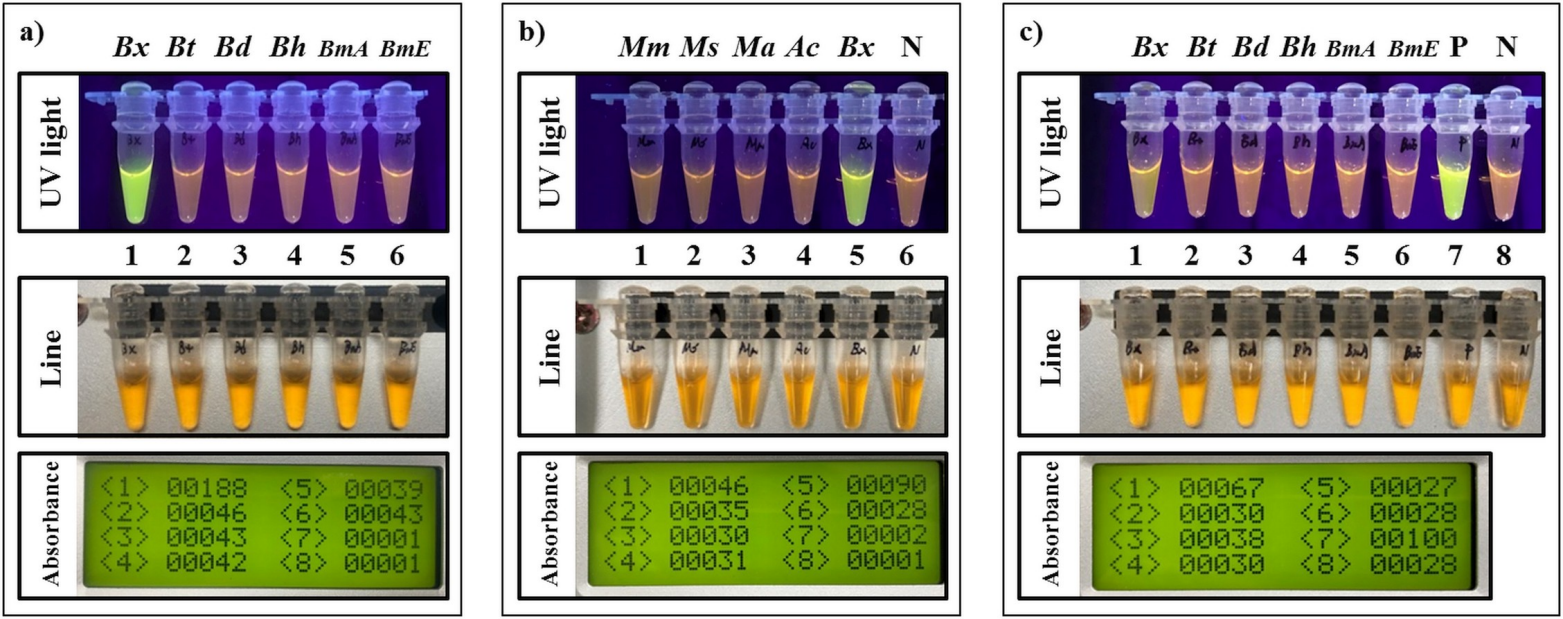
Three types of specificity tests of the RPA assay for *B*. *xylophilus*. a) The specificity test of the RPA assay using 6 ng of pure gDNA from *B*. *xylophilus* (*Bx*) and five *Bursaphelenchus* spp.: *B*. *thailandae* (*Bt*), *B*. *doui* (*Bd*), *B*. *hylobianum* (*Bh*), *B*. *mucronatus* Asia type (*BmA*), and *B*. *mucronatus* Europe type (*BmE*) which have strong genetic relationships with *B*. *xylophilus*. b) The specificity test of the RPA assay on organisms: *Matsucoccus matsumurae* (*Mm*), *Monochamus saltuarius* (*Ms*), *Monochamus alternatus* (*Ma*), and *Anoplophora chinensis* (*Ac*) which have weak genetic relationships with *B*. *xylophilus*. c) The specificity test of the RPA assay using spiked pinewood from healthy pine tree with pure gDNA from nematodes. The absorbance and UV light represent the detection results of the POID and the UV light, respectively. P and N represent the positive control using pure gDNA from *B*. *xylophilus* and the negative control using lysate from healthy pine tree extracted using the DAP buffer, respectively. The line numbering is matched with the absorbance numbering in each panel. The absorbance value of the zero point in the RPA assay was set to 50. Therefore, values of less than 50 measured by the POID are considered not specifically amplified.

The results for the estimation of the zero absorbance points (offset value) of the RPA assay measured by the portable optical isothermal device (POID) were set to exclude data caused by the non-specific amplification of the RPA reaction. In this experiment, the repeated RPA reactions using lysates of the healthy pine tree, non-gDNA, and non-primer reactions showed absorbance values of less than 50 (Fig 3). Therefore, further experiments were carried out on the assumption that absorbance values below 50 were not the result of specific amplification from the gDNA of *B*. *xylophilus* that targets the RPA assay. The specificity test of the RPA assay using the pure gDNA of five *Bursaphelenchus* spp. which have strong genetic relationships with *B*. *xylophilus* [14] did not show non-specific amplification in the reaction. Positive amplification was only shown with pure gDNA from *B*. *xylophilus* (Fig 4A).

In addition, no cross‐reactions were detected using species which have low genetic relationships with *B*. *xylophilus* (Fig 4B). Lastly, as with using the pure gDNA of nematodes, the result of the RPA assay using the spiking pure gDNA of nematodes with the pinewood of a healthy pine tree showed that a positive reaction was only detected in the spiked sample with the pure gDNA of *B*. *xylophilus* (Fig 4C). These results indicate that the RPA assay for the PWN is specific for *B*. *xylophilus* and could distinguish *B*. *xylophilus* from nematodes with strong genetic relationships, even for the samples mixed with pinewood.

### Limit of detection test of the RPA assay

**Fig 5:**
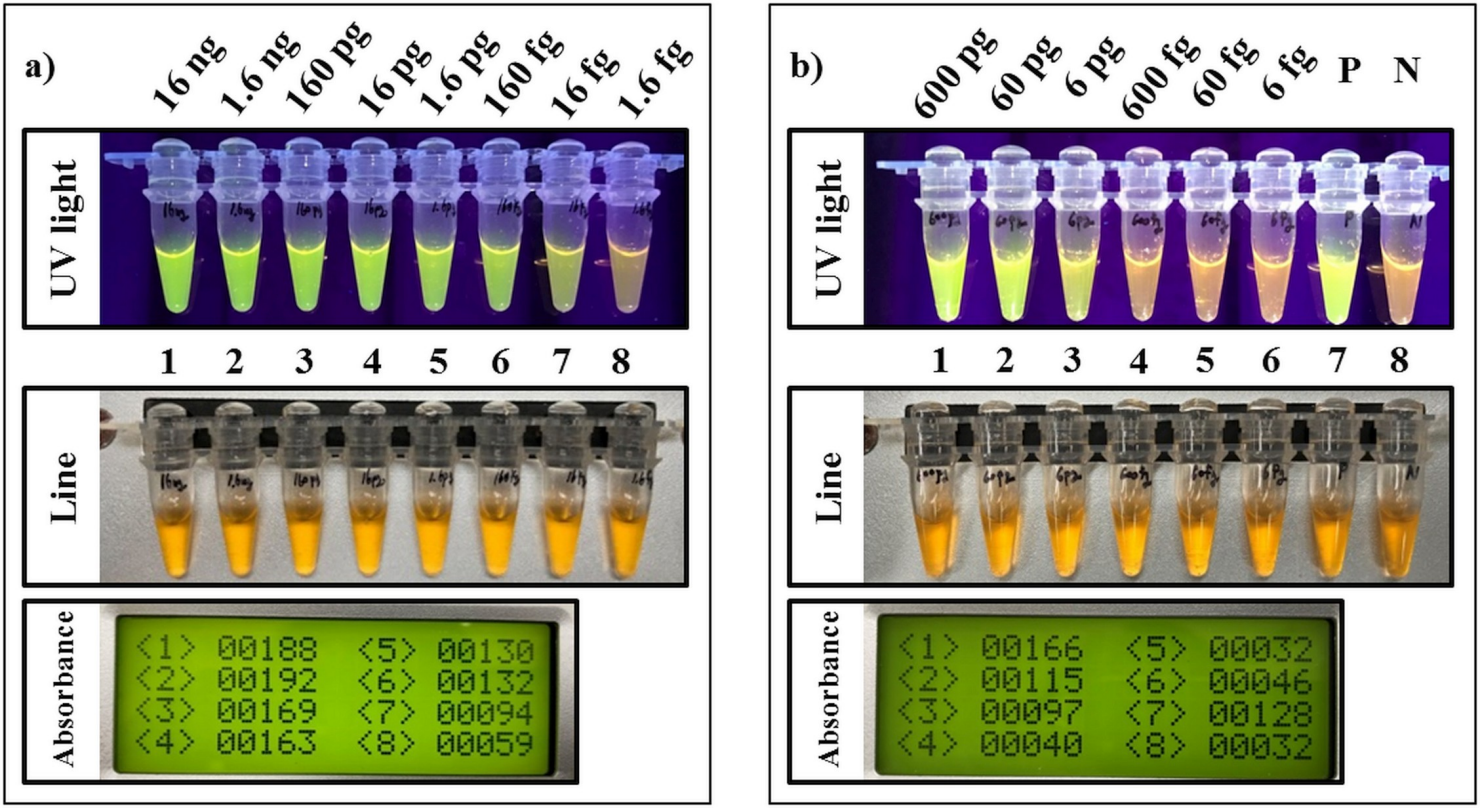
The evaluation of the limit of detection (LOD) in the RPA assay for *B*. *xylophilus*. First, a) The RPA assay used a ten-fold serial dilution of pure gDNA of *B*. *xylophilus* to measure the LOD under conditions without any interference except pure gDNA. Second, b) For the measurement for the pinewood sample LOD, RPA assay used the lysates were made by healthy pinewood mixed with the pure gDNA of *B*. *xylophilus*, which was then extracted by DAP buffer to measure the LOD under conditions with all interferences come from the pinewood with DAP buffer. P and N represent the positive control using pure gDNA from *B*. *xylophilus* and the negative control using healthy pine tree lysate extracted by DAP buffer. The line numbering is matched with absorbance numbering in each panel.

The results of using the pure gDNA of *B*. *xylophilus* showed that less than 1.6 fg of gDNA could not be reliably detected by UV light, but it could be measured precisely by POID. The results also showed an improvement in LOD when using the pure gDNA compared to the previous RPA assay using IGS primers [24] (Fig 5A and S3 Appendix). The measured the pinewood sample LOD value of the RPA assay, which reflected all disturbances, was higher than when using pure gDNA (Fig 5B). This result may be due to the inhibition of the RPA reaction by components such as humic acid, ethanol, polysaccharide, and polyphenol which were contained in pinewood [25], or due to the presence of the DAP buffer which formed high-alkaline solutions.

Nevertheless, the RPA assay detected up to a final concentration of 6 pg of gDNA from *B*. *xylophilus* in the wood lysate extracted by the DAP buffer, even when mixed with pinewood. However, it is very difficult or impossible to count the exact number of *B*. *xylophilus* in pinewood chips since there have debris (cut by a drill or natural dead) or eggs from *B*. *xylophilus*. Considering this condition, we only estimated that the LOD from individuals of *B*. *xylophilus* per 100 mg of pinewood chips by comparing pinewood sample LOD of RPA assay in this study and results from previous researches.

The real-time PCR research for detecting PWN revealed that a single adult of *B*. *xylophilus* has approximately 50–500 ng of gDNA [19]. Therefore, the results of the pinewood sample LOD might indicate that even if only one individual adult of *B*. *xylophilus* was included in about 100 mg of pinewood, the RPA assay might be able to amplify the ITS2 fragment of *B*. *xylophilus*. More practically, the pinewood sample LOD of the RPA assay is ten times lower compared with the RT-LAMP assay using the pinewood sample directly (could be amplified in over ten individuals of *B*. *xylophilus* per 100 mg of pinewood) [26] without considering the dilution factor.

### The detection of residual environmental DNA from *B*. *xylophilus* in pinewood

**Fig 6:**
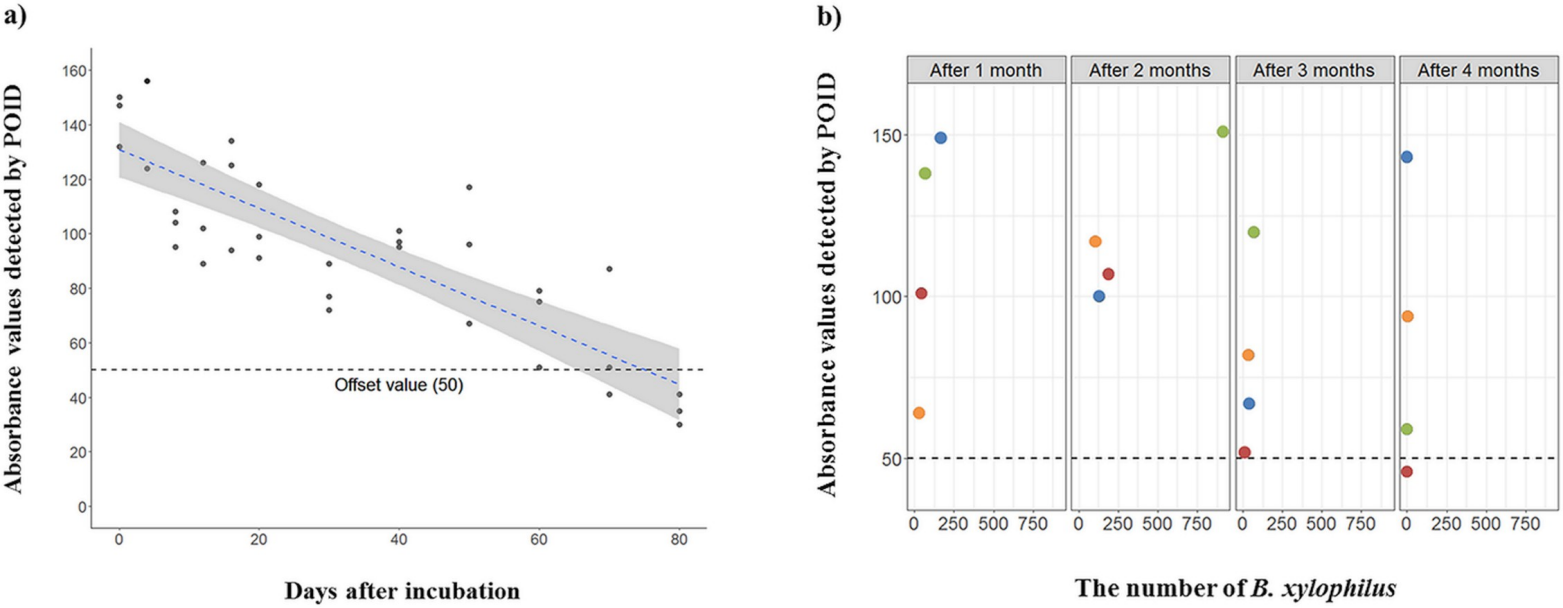
Detection of environmental DNA (eDNA) from *B*. *xylophilus* in pinewood logs. a) Summary data of the absorbance values of the RPA assay measured using pinewood logs which were incubated at 50°C and 40% humidity. The blue dashed line and the grey background represents the regression model line and 95% confidence interval, respectively. b) Evaluation of the RPA assay for the remaining gDNA from *B*. *xylophilus* in pine trees placed in natural conditions. The RPA assay was conducted once a month for four months and the number of *B*. *xylophilus* per 10 g of pinewood for each log was assessed for every RPA assay. Black dashed lines and colored circles in each panel represent offset values and each log, respectively.

Our surrounding environments contain a large amount of gDNA debris originating from various organisms through carcasses, excretion, or ecdysis (the specific growing process in hexapods, nematodes, and reptiles). In recent years, several studies have reported that environmental DNA (eDNA) may be used for the identification of various organisms, especially fish, because eDNA is weakened and destroyed by UV and high temperatures [27,28]. In addition, these studies revealed that the total amount of eDNA from one species in the environment is highly dependent on their population density [29]. According to these studies, we tested how long the eDNA from *B*. *xylophilus* remained in pinewood via an RPA assay under the assumption that eDNA from *B*. *xylophilus* could remain in logs of PWN-infected pine trees under various conditions. First, artificial conditions were designed for estimating the degree of eDNA destruction in pinewood after nematode death due to high-temperature conditions over a short period [30]. As time passed, the RPA amplification levels showed gradually decreasing absorbance values through POID, indicating that eDNA originating from *B*. *xylophilus* was degraded by high-temperature conditions. Four days after incubation, 10 g of pinewood from each log was extracted via the Baermann funnel method to identify whether live *B*. *xylophilus* inhabit the logs. However, live *B*. *xylophilus* were not extracted. After 70 days, one out of three RPA reactions had not been positively detected by POID. However, the eDNA remained sufficient enough to amplify the RPA assay from 4 to 70 days after incubation at 50°C and 40% humidity (Fig 6A and S4 Appendix). The simple artificial temperature conditions did not account for the effects of other fungi, bacteria, and UV, which are factors in the destruction of eDNA in the environment [27]. Therefore, in the following experiment, the logs of PWN-infected pine trees were placed under natural conditions as much as possible. The populations of *B*. *xylophilus* in each log varied, and termites were present, and mold grew in some logs which might have affected the residual eDNA in the pine tree logs. Otherwise, the eDNA from *B*. *xylophilus* was detected by the RPA assay for up to 120 days in natural conditions (Fig 6B and S5 Appendix). Considering these results, the RPA assay showed the possibility of their use for epidemiological surveys using dead pine tree logs even when living *B*. *xylophilus* are not present, but there is sufficient eDNA which can be amplified by an RPA assay in the field.

## Conclusions

In this study, the RPA assay, which comprises DAP buffer and newly designed ITS2 primers for the detection of PWN using pinewood samples, has been improved in three ways. First, this method is 100 times more sensitive to the detection of PWN than previous RPA assay conducted using IGS primers for assessing the LOD. Second, by using the POID, the RPA assay can distinguish the positives and negatives precisely by measuring the levels of absorbance in 25 minutes. Lastly, experimental procedures were composed of three steps (quick gDNA extraction, RPA amplification, and measuring absorbance levels), that aid users in working in the field. Together, the newly designed RPA assay with the POID is relatively acute, rapid, and can easily confirm results compared with previous molecular methods for the detection of PWN, indicating that RPA with POID can potentially be used as an on-site diagnostic method for PWD.

## Supporting information

S1 Appendix The feature of portable optical isothermal device. (TIF)

S2 Appendix Duration test for mater mixture of RPA assay. P and N represent positive control (pure gDNA of *B*. *xylophilus*) and negative control (lysate of healthy pine tree), respectively.
(TIF)

S3 Appendix End-point absorbance value comparison between the amplification of ITS2 and IGS from pure gDNA of *B*. *xylophilus*. a) Limit of detection in RPA assay using ITS2 primer. b) Limit of detection in RPA assay using IGS primer.
(TIF)

S4 Appendix Detection of residual environmental DNA from *B*. *xylophilus* in pinewood under 50°C condition. P and N represent positive control (pure gDNA of *B*. *xylophilus*) and negative control (lysate of healthy pine tree), respectively.
(TIF)

S5 Appendix Detection of residual environmental DNA from *B*. *xylophilus* in pinewood in natural condition. P and N represent positive control (pure gDNA of *B*. *xylophilus*) and negative control (lysate of healthy pine tree), respectively.
(TIF)
