# Supplementary figures and images for: Point-of-care diagnostic (POCD) method for detecting Bursaphelenchus xylophilus in pinewood using recombinase polymerase amplification (RPA) with the portable optical isothermal device (POID)
Source: PLoS One. 2020 Jan 14;15(1):e0227476. doi: 10.1371/journal.pone.0227476 (PMC6959569; doi:10.1371/journal.pone.0227476)

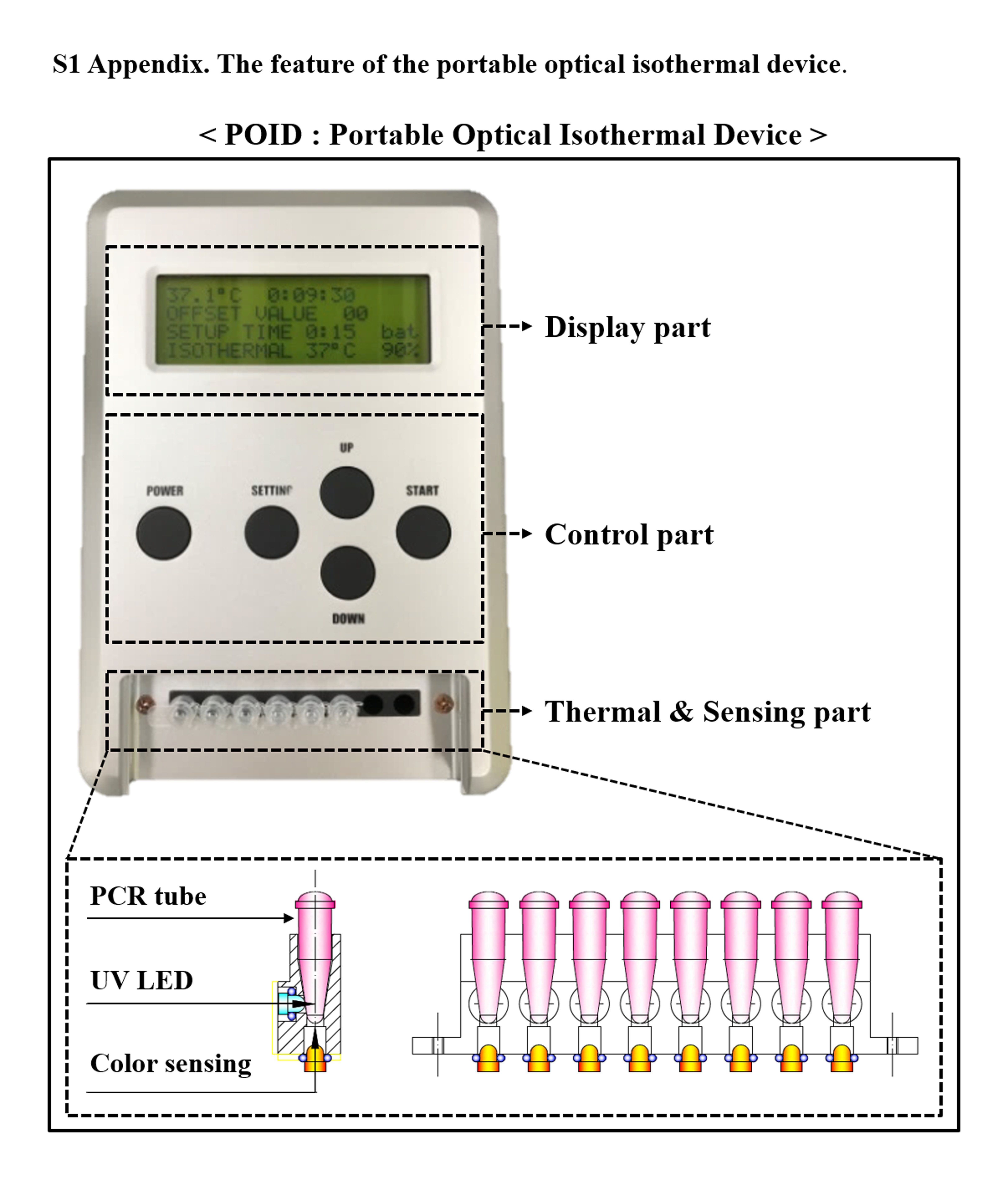

Supplement: S1 Appendix — (TIF) [file pone.0227476.s001.tif]

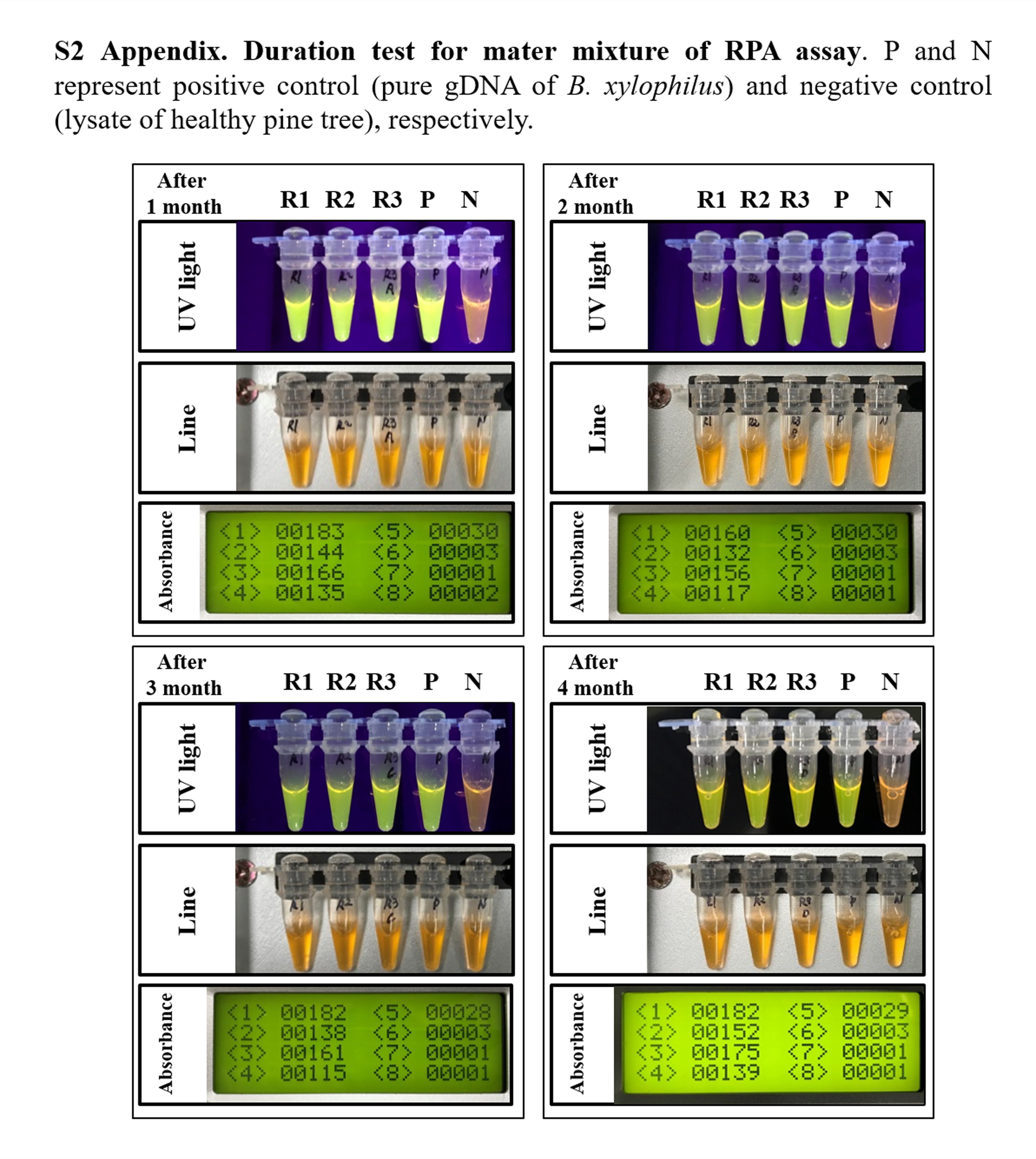

Supplement: S2 Appendix — P and N represent positive control (pure gDNA of B. xylophilus) and negative control (lysate of healthy pine tree), respectively. (TIF) [file pone.0227476.s002.tif]

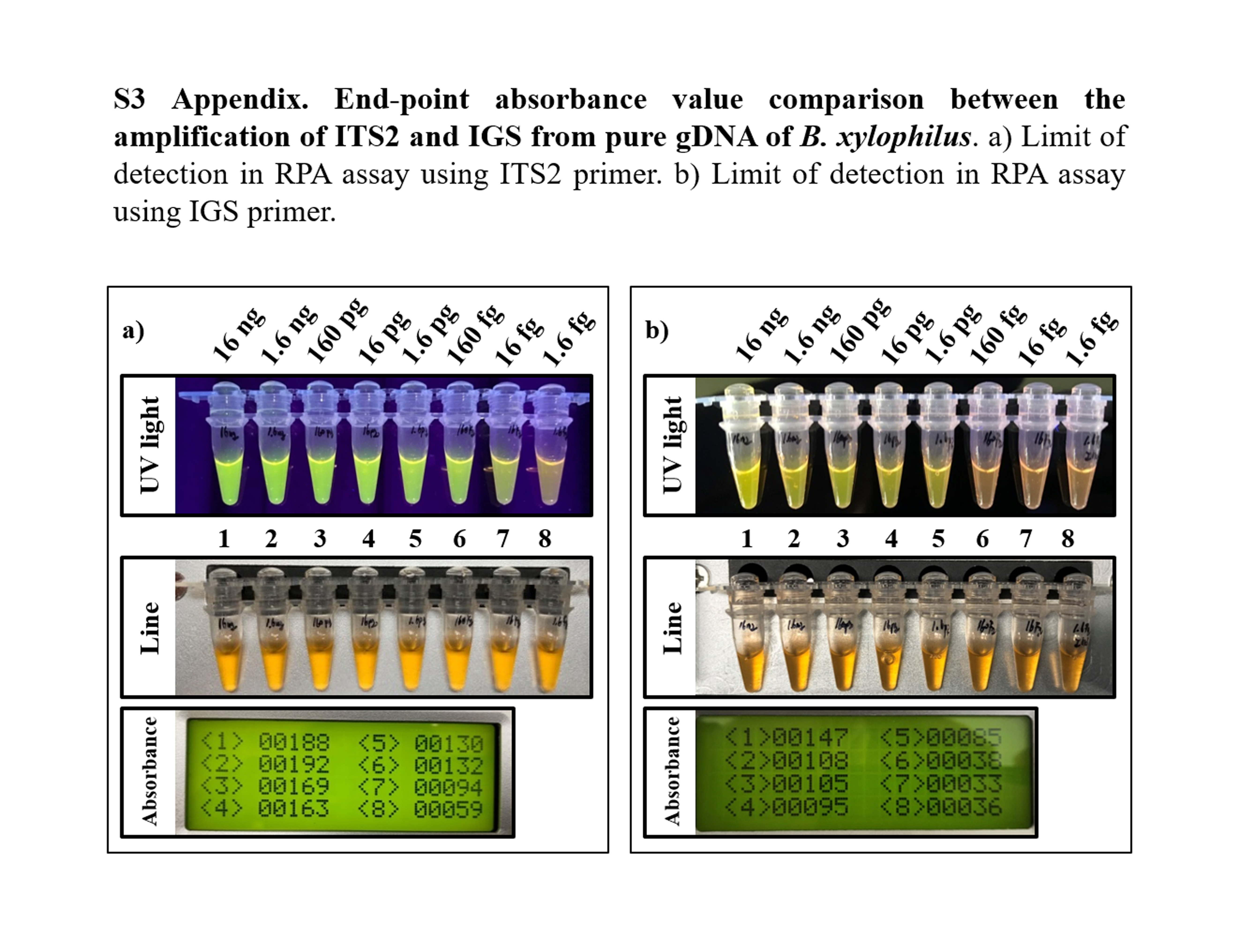

Supplement: S3 Appendix — a) Limit of detection in RPA assay using ITS2 primer. b) Limit of detection in RPA assay using IGS primer. (TIF) [file pone.0227476.s003.tif]

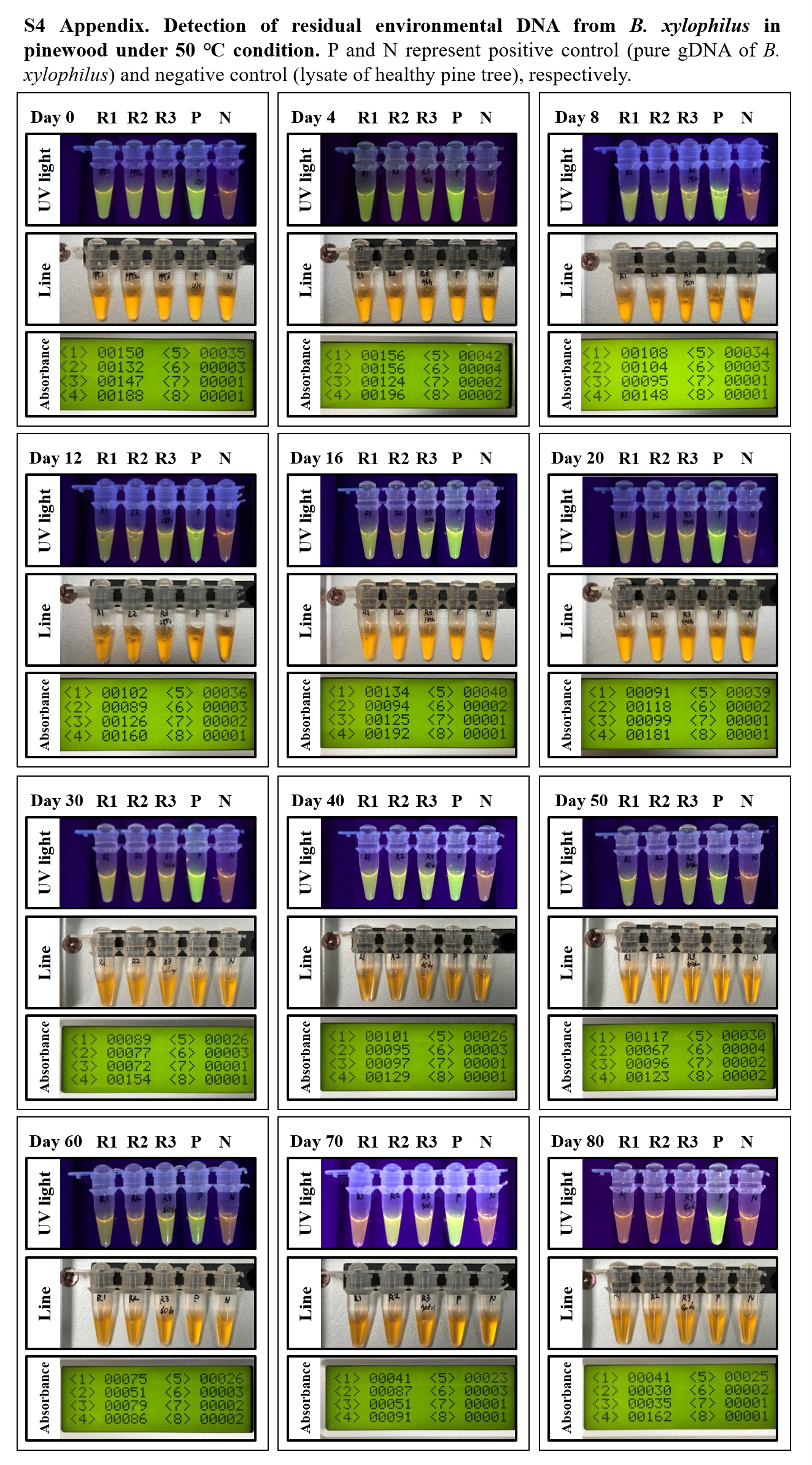

Supplement: S4 Appendix — P and N represent positive control (pure gDNA of B. xylophilus) and negative control (lysate of healthy pine tree), respectively. (TIF) [file pone.0227476.s004.tif]

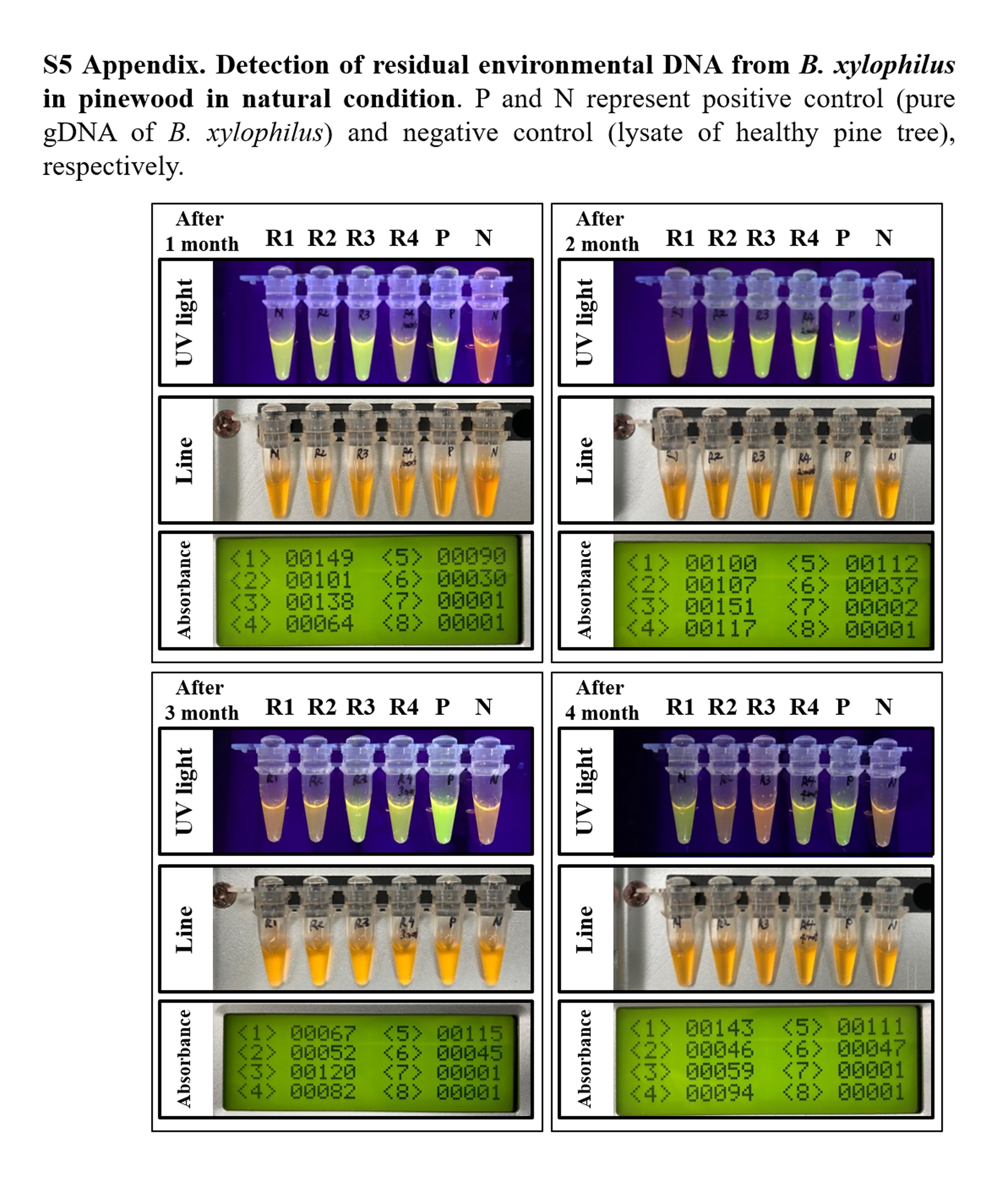

Supplement: S5 Appendix — P and N represent positive control (pure gDNA of B. xylophilus) and negative control (lysate of healthy pine tree), respectively. (TIF) [file pone.0227476.s005.tif]
